# Effect of sneezing on the flow around a face shield

**DOI:** 10.1063/5.0031150

**Published:** 2020-12-08

**Authors:** Fujio Akagi, Isao Haraga, Shin-ichi Inage, Kozaburo Akiyoshi

**Affiliations:** 1 Faculty of Engineering, Fukuoka University, 8-19-1 Nanakuma, Jyounan-ku, Fukuoka, Japan; 2 Department of Anesthesiology, Faculty of Medicine, Fukuoka University, 7-45-1 Nanakuma, Jyounan-ku, Fukuoka, Japan

## Abstract

A flow analysis around a face shield was performed to examine the risk of virus infection when a medical worker wearing a face shield is exposed to a patient’s sneeze from the front. We ensured a space between the shield surface and the face of the human model to imitate the most popularly used face shields. In the present simulation, a large eddy simulation was conducted to simulate the vortex structure generated by the sneezing flow near the face shield. It was confirmed that the airflow in the space between the face shield and the face was observed to vary with human respiration. The high-velocity flow created by sneezing or coughing generates vortex ring structures, which gradually become unstable and deform in three dimensions. Vortex rings reach the top and bottom edges of the shield and form a high-velocity entrainment flow. It is suggested that vortex rings capture small-sized particles, i.e., sneezing droplets and aerosols, and transport them to the top and bottom edges of the face shield because vortex rings have the ability to transport microparticles. It was also confirmed that some particles (in this simulation, 4.4% of the released droplets) entered the inside of the face shield and reached the vicinity of the nose. This indicates that a medical worker wearing a face shield may inhale the transported droplets or aerosol if the time when the vortex rings reach the face shield is synchronized with the inhalation period of breathing.

## I. INTRODUCTION

The COVID-19 pandemic has considerably infected the global population, and the threat continues. Moreover, the number of infected people exceeds the number of medical staff and medical equipment in several countries; therefore, many infected persons and patients with cases unrelated to COVID-19 cannot receive adequate medical treatment.^1,2^ To maintain the quality of medical treatment in such situations, infection prevention measures for medical staff are extremely important.^3,4^ Face shields, which cover the face with a clear plastic screen, and medical surgical masks are actively being used by medical staff to prevent inhalation of virus-laden droplets that spread by breathing, coughing, and sneezing of an infected person.^5–7^ Recently, the number of people using face shields as a substitute for face masks has been increasing in schools, universities, restaurants, and service businesses. Factors driving this increased adoption include the benefits of being able to see facial expressions, ease of hearing, reusability when washed and disinfected properly, and increased comfort compared to regular masks. However, there is one concern with this. As face shields and masks were originally assumed to prevent the spread of our own droplets, it may not be effective in preventing infection when used in any other way.

Numerous studies have been conducted to determine the effective means to prevent infection.^6–14^ Their results show that some of the preventive measures that were previously considered to be effective have not been fully effective in stopping the virus. For example, it has been suggested that face shields and masks equipped with exhalation valves cannot provide sufficient prevention of infection.^10–12^ Verma *et al.* conducted a flow visualization around a face shield and a mask to evaluate their performance in preventing the spread of aerosol-sized droplets when an infected person uses them for the purpose of protecting others.^10^ They observed aerosols leaking into the air through the gaps in face shields and masks; they concluded that it may be desirable to use a high-quality cloth or surgical mask with a plain design. On the contrary, this also applies when wearing a face shield or mask for the purpose of preventing infection from infected persons. For example, if a medical worker wearing a face shield is exposed to droplets from an infected person’s breath, cough, or sneeze in front of the face, large droplets may attach to the shield’s surface. However, small droplets may move with the flow and be drawn in through the space between the face and the face shield.^12,15–20^ The airflow caused by sneezing has a particularly high velocity. There is a high possibility that droplets will flow into the shield owing to the entrainment flow at the edge of the face shield. However, the details of the airflow around the face shields and their effectiveness in preventing infection when a shield wearer is exposed to a patient’s sneeze have not yet been clarified.

In this study, flow simulation around the face shield was performed to investigate the risk of infection for medical staff wearing the face shield when the person was exposed to a patient’s sneeze from the front.

## II. NUMERICAL METHOD

### A. Computational domain and grid

**FIG. 1.**
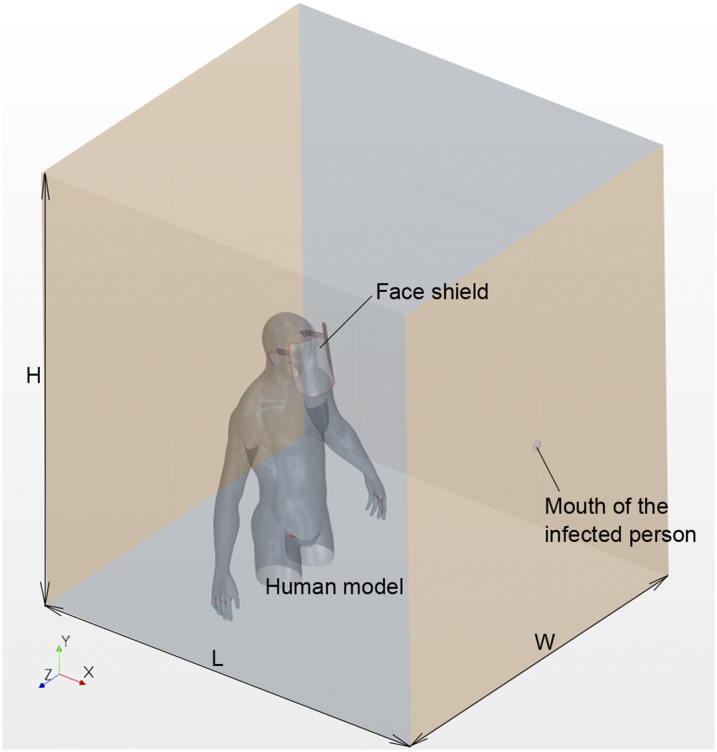
Schematic of the computational domain.

**FIG. 2.**
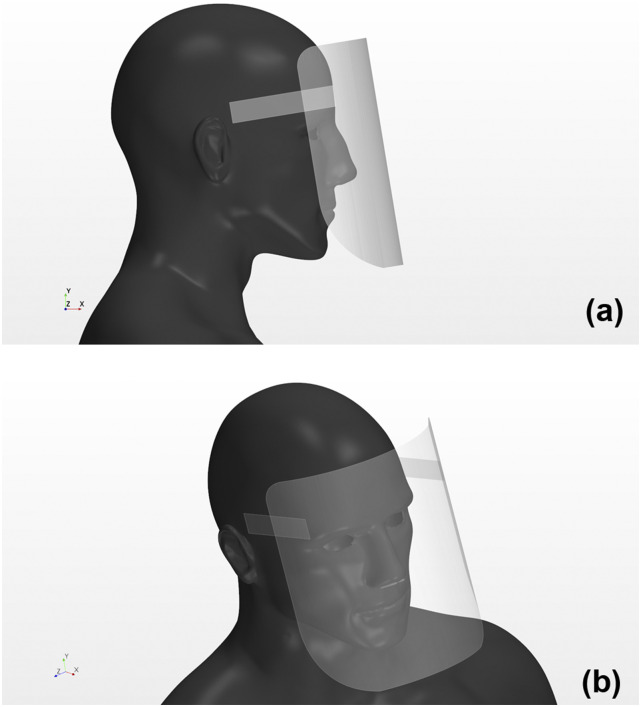
Human model wearing a face shield. The face shields were targeted at the most popularly used shields. (a) Side view; (b) diagonal top view.

Figure 1 depicts a schematic of the computational domain used in the present simulation. The dimensions of this domain are 2 × 2 × 2.5 m^3^ in the streamwise (L), vertical (H), and width (W) directions, respectively. A life-sized human model wearing a face shield was placed at the center of the computational domain. Figure 2 shows the human model with a face shield on (side view and diagonally top view). We ensured a space between the shield surface and the face of the human model to imitate the most popularly used face shields. Most face shields used in the medical field are designed with no space between the upper edge of the shield surface and the user’s face. The evaluation of this type of shield can be conducted by referring to the flows at the sides and the lower end of the shield used in this simulation. The distance between the face shield and the face was set to an average of 25 mm. This fitting condition is close to reality. The average distance between medical staff and infected patients during treatment and diagnosis was estimated to be ∼1 m.^16,18^ Therefore, the distance between the mouth of the infected person (40 mm in diameter) and the surface of the face shield facing the infected person was set to 1 m in the simulation.

**FIG. 3.**
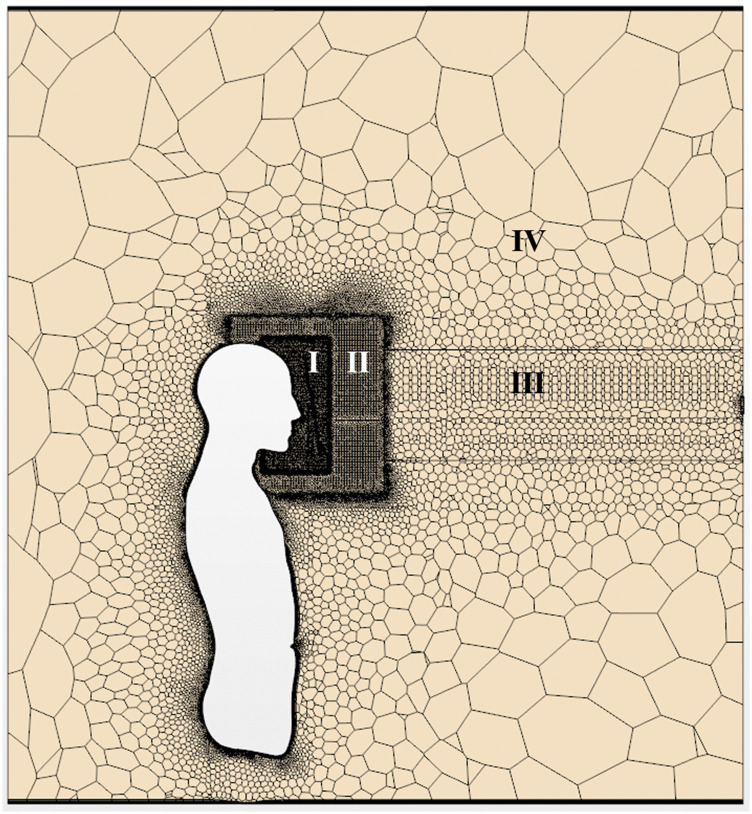
Computational grid on a vertical section in the center of the domain. The computational mesh was generated using polyhedral non-uniform structured grids. The computational domain was divided into four regions (regions I, II, III, and IV).

The computational grids used in the simulation are illustrated in Fig. 3. The computational mesh was generated using polyhedral, nonuniform-structured grids. As shown, the computational domain was divided into four regions (regions I, II, III, and IV). Specifically, the grid was clustered in region I, which included the face of the human model and face shield surface. Moreover, the preliminary estimation of the Kolmogorov length scale of the present sneezing flow was ∼3.5 × 10^−4^ m; therefore, this fine region consisted of grids of size of 8.0 × 10^−4^ m (approximately double the Kolmogorov length scale). The minimum size of grids on the surface of the shield and face was set such that it was sufficiently small to satisfy the condition y^+^ < 1. In the region outside region I, the size of the grid was enlarged gradually following a geometric progression. The estimated number of cells in regions was 6 099 234 for region I, 5 343 843 for region II, 70 242 for region III, and 1 906 316 for region IV. Therefore, the total number of cells was ∼13.4 × 10^6^.

### B. Boundary conditions

**FIG. 4.**
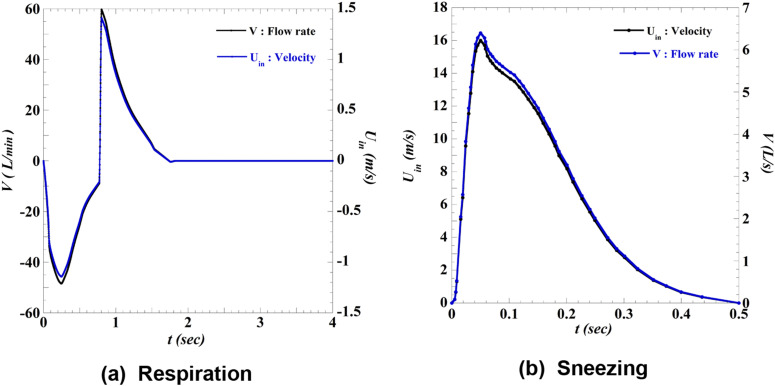
Waveform of airflow velocity and flow rate by respiration. Both waveforms were modeled considering a typical adult male. (a) Flow rate and velocity waveform of the respiration; (b) flow rate and velocity waveform of sneeze.

Figure 4 plots the velocity and flow waveforms of the airflow by respiration and sneezing.^9,19^ In Fig. 4(a), a negative velocity period indicates the inhalation period, whereas a positive velocity period indicates the exhalation period. This velocity condition was applied to two elliptical inlet boundary surfaces (a major diameter of 20 mm and a minor diameter of 10 mm) located at the nose of the human model. In short, this simulation assumes nasal-only respiration. The sneezing velocity condition [Fig. 4(b)] was applied to the inlet boundary surface, which simulates the mouth of an infected person. Because the temperature of the air in the mouth is ∼32 °C,^11,17^ the temperature of the outward airflow owing to exhaling and sneezing is generally different from that of the outside air. Furthermore, because this temperature difference produces an effect of buoyancy, we conducted simulations to consider this effect. Herein, we set the air temperature at the inlet boundary as 32 °C (305 K) and the outside temperature as 27 °C (300 K).

The no-slip condition was applied to the face shield surface and the human model. Specifically, in the computational domain, the aforementioned condition was applied to all boundaries except to the backward boundary of the human model, for which the Neumann condition was applied such that the gradient of the physical value was zero.

### C. Flow solver

In the present simulation, a large eddy simulation was conducted to simulate the vortex structure generated by the sneezing flow near the face shield. The code solved the filtered three-dimensional compressible Navier–Stokes equations using a fully implicit scheme with the finite-volume method.^20^ The convection flux was evaluated using the bounded central-difference scheme. We found that this scheme turned into a first-order upwind scheme when the convection boundedness criterion was not satisfied. In other cases, it was similar to the central-difference scheme, which is second-order accurate. The sub-grid eddy viscosity was modeled using the Wall-Adapting Local-Eddy Viscosity (WALE).^21^

To obtain a time-accurate solution, the second-order backward difference is applied at each time step until the residual of the solution becomes less than 1 × 10^−6^. The time step size of the implicit scheme was set to 5 × 10^−4^ s, corresponding to a Courant number of 1.0. The computational fluid dynamics code StarCCM+ (version 12.06.011) was employed, and the computations were performed on 2 intel-Xeon (E5-2699 v4: the total number of cores = 44) processors. The computational time consumed by our simulation experiment was ∼1 week. Note that the results of other simulations of coaxial jets using the computational conditions and solver presented above were in good agreement with the experimental results.^22^

## III. RESULTS AND DISCUSSION

### A. Flow by respiration

**FIG. 5.**
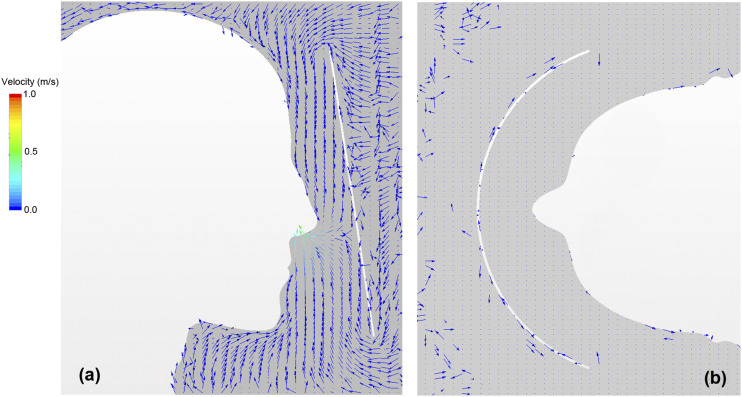
Velocity vector distribution of airflow around the face shield at the time of the maximum suction flow rate of respiration (t = 0.3 s). The velocity vector distribution along (a) the vertical cross section and (b) the horizontal cross section.

We conducted simulations to visualize the effect of human respiration on the airflow around the face shield. Figure 5 depicts the velocity vector distribution of the airflow around the face shield at the time of the maximum suction flow rate during inhalation (t = 0.3 s). Figure 5(a) depicts the vertical projections of the velocity vectors along the vertical cross section. At the top and bottom edges of the face shield, a low-velocity flow (less than 10 cm/s) from the outside to the inside was generated by the suction air. Figure 5(b) shows the horizontal projections of the velocity vectors in the horizontal cross section. Figure 5(b) shows that no horizontal flow is generated in this cross section. This indicates that the flow in the shield enters from the upper and lower ends of the shield and moves toward the nose.^23,24^

**FIG. 6.**
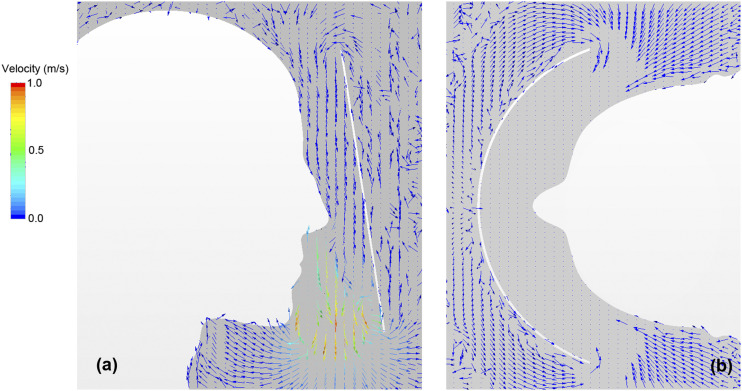
Velocity vector distribution of airflow around the face shield at the time of maximum exhalation flow rate of respiration (t = 1.0 s). The velocity vector distribution along (a) the vertical cross section and (b) the horizontal cross section.

Figure 6 maps the velocity vector distribution around the face shield at the time of the maximum exhalation flow rate of respiration (t = 1.0 s). Specifically, Fig. 6(a) shows the vertical projections of the velocity vectors along the vertical section. A high-velocity flow (more than 1 m/s) from the inside of the shield to the outside was generated by the exhalation flow at the lower end of the shield, whereas a low-velocity flow from the outside of the shield to the inside was generated at the upper end of the shield. Figure 6(b) shows the velocity vector distribution along the horizontal cross section. It can be observed that a flow was generated from the sides of the shield into the inside, and this flow turned vertically after the inflow. Therefore, the flow in the space between the face shield and the face was observed to vary with human respiration.

**FIG. 7.**
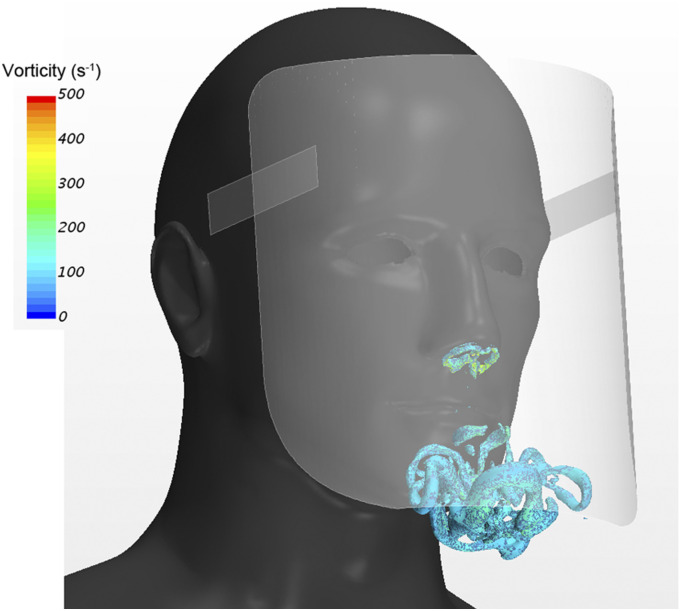
Three-dimensional vortex structure generated by exhalation. The vortex structure is represented by the isosurfaces of the second invariant of the velocity gradient tensor (Q-criterion). Multimedia view: https://doi.org/10.1063/5.0031150.110.1063/5.0031150.1

Figure 7 (Multimedia view) illustrates the three-dimensional vortex structure generated by exhalation. The vortex structure is represented by the isosurfaces of the second invariant of the velocity gradient tensor (Q-criterion). The high-velocity flow caused by the exhalation generates vortex ring structures. Subsequently, this vortex ring becomes unstable and is deformed in three dimensions. It is suggested that the aerosols emitted during exhalation may be transported by these vortex rings because they have the ability to transport microparticles.^21,22^

### B. Flow by sneezing

We conducted a flow simulation under the condition that a medical staff wearing a face shield is exposed to a sneeze from the front direction with its source at a distance of 1 m. To simulate the risk of infection in medical staff, the time of the staff’s inhalation should be synchronized with the time when droplets emitted from sneezing reach the surroundings of the face shield. Therefore, in this simulation, the start time of staff’s inhalation was set to 0.25 s after the start of sneezing, considering the time spent on the arrival of the droplets.

**FIG. 8.**
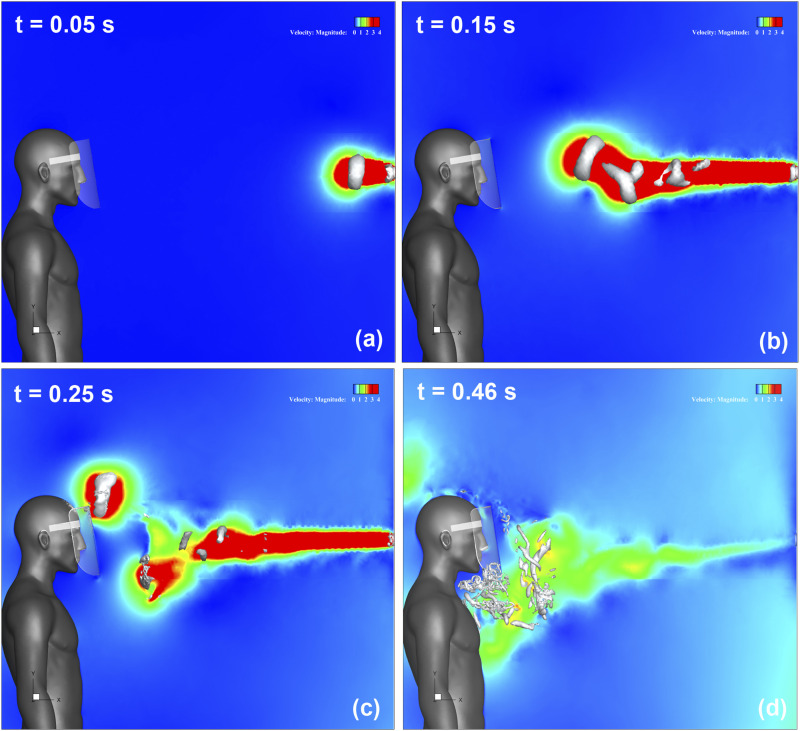
Streamwise velocity distribution along the vertical cross section in the center of domain and the three-dimensional vortex structure. The color contours represent the magnitude of the velocity, and the isosurfaces represent the vortex structures. (a) After 0.05 s from the start of sneezing; (b) after 0.15 s; (c) after 0.25 s; (d) after 0.46 s. Multimedia view: https://doi.org/10.1063/5.0031150.210.1063/5.0031150.2

Figure 8 (Multimedia view) depicts the streamwise velocity distribution and evolution of the three-dimensional vortex structure. Figure 8(a) (Multimedia view) represents that t = 0.05 s after the start of sneezing. The sneeze generates a jet-like high-velocity flow downstream of the mouth of the infected person, and a vortex ring is generated. Furthermore, this vortex ring moves forward by its self-induced velocity and changes direction upward, as shown in Fig. 8(b) (Multimedia view). Subsequently, it reaches the top of the face shield at t = 0.25 s, as shown in Fig. 8(c) (Multimedia view). At this time, a high-velocity region owing to the rotation of the vortex ring can be observed at the upper end of the shield. Behind this vortex ring, several other vortex rings were generated [Fig. 8(b)] (Multimedia view). These rings gradually moved downward [Fig. 8(c) (Multimedia view)] and reached the lower end of the shield as they broke down [Fig. 8(d)] (Multimedia view). The flow in the vicinity of the throat of the shield user is strongly disturbed by this breakdown of the vortex rings.

**FIG. 9.**
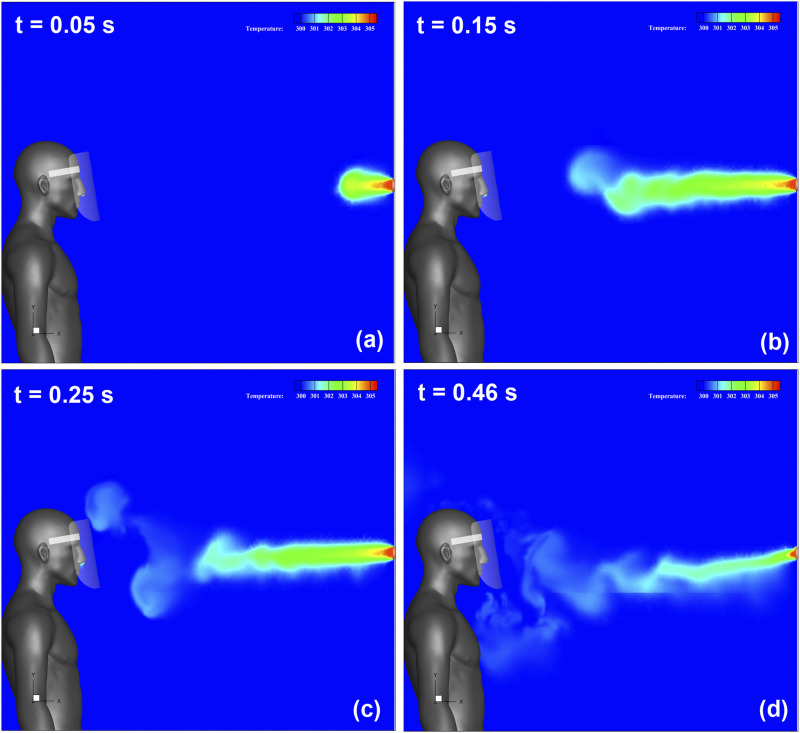
Temperature distribution along the vertical cross section in the center of the domain. The initial temperature of the sneeze set to 32 °C (305 K), and the temperature of the ambient air set to 27 °C (300 K). (a) After 0.05 s from the start of sneezing; (b) after 0.15 s; (c) after 0.25 s; (d) after 0.46 s. Multimedia view: https://doi.org/10.1063/5.0031150.310.1063/5.0031150.3

In the present simulation, it was observed that the leading vortex ring tended to move upward and not toward the left or right side. This could be because of the effect of buoyancy owing to the temperature difference between the sneezing and the ambient air in the domain. Figure 9 (Multimedia view) depicts the temperature distribution along the vertical cross section corresponding to each time in Fig. 8 (Multimedia view). It shows that the temperature in the vortex core is ∼3 °C higher than the ambient temperature because the leading vortex ring is generated by rolling up the shear layer of the sneeze flow [Fig. 9(a)] (Multimedia view), and the difference in temperature gradually decreases with the movement of the vortex ring [Figs. 9(b) and 9(c)] (Multimedia view). This temperature difference acts as a buoyant force on the leading vortex ring, which makes it easier to move upward. Conversely, the backward vortex rings moved downward despite the same temperature difference as the leading vortex rings. This is because the induced velocity of the leading vortex ring affects the backward vortex rings, and this velocity is larger than the rising velocity caused by buoyancy.

**FIG. 10.**
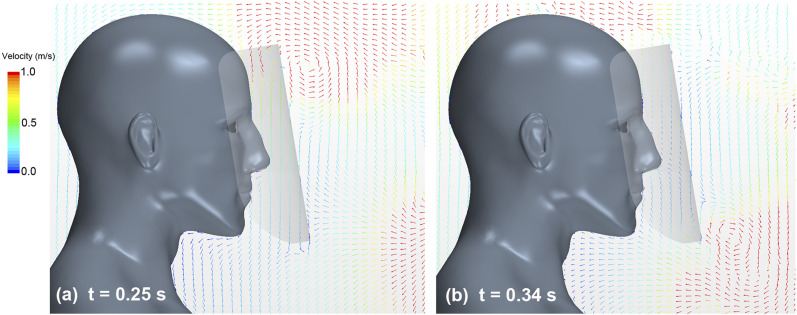
Velocity vector distribution of airflow along the vertical cross section at the center of the domain. The arrows and the colors represent velocity vectors and the magnitudes of the velocities, respectively. (a) After 0.25 s from the start of sneezing; (b) after 0.34 s. Multimedia view: https://doi.org/10.1063/5.0031150.410.1063/5.0031150.4

Figure 10 (Multimedia view) depicts the distribution of velocity vectors along the vertical cross section at t = 0.25 s and 0.34 s. As shown in Fig. 10(a) (Multimedia view), when the leading vortex ring reached the top of the shield, a high-velocity flow (more than 1 m/s) was generated toward the inside of the shield owing to the rotation of the vortex ring. Subsequently, as illustrated in Fig. 10(b) (Multimedia view), we found that when the following vortex rings reached the lower end of the shield, the flow (velocity was less than 0.5 m/s) was generated toward the inside of the shield by the vortex rings.

**FIG. 11.**
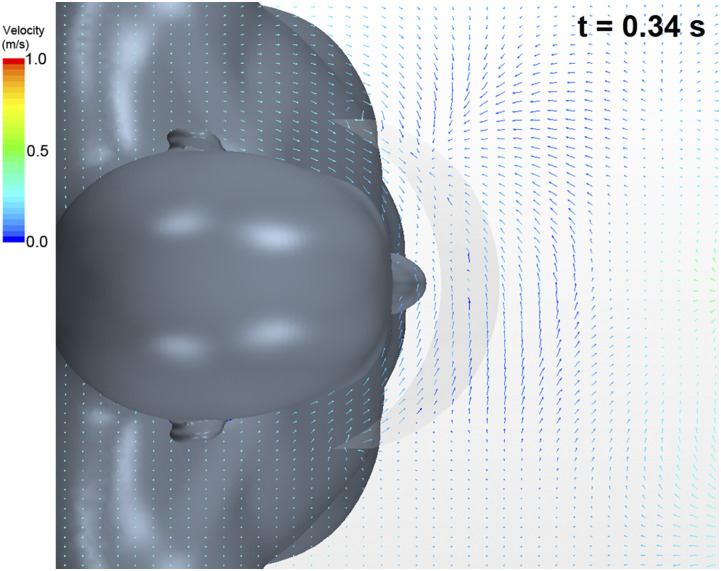
Velocity vector distribution of airflow along the horizontal cross section. The arrows represent velocity vectors, and the colors represent the magnitude of the velocity.

Figure 11 maps the simultaneous velocity vector distribution along the horizontal cross section as compared to Fig. 10(b) (Multimedia view). We observed that a flow was generated from the sides of the shield toward its inside (at a velocity less than 0.5 m/s). The magnitude of this inflow velocity was highest at this instant.

As discussed earlier, the jet-like flow owing to sneezing generates vortex rings, which reach the top and bottom edges of the shield and form a high-velocity entrainment flow. Moreover, because these vortex rings have the ability to transport microparticles, it is assumed that the vortex rings generated by the sneeze could capture microsized droplets and transport them to the face shield. If the transported droplets are caught in the entrainment flow formed by vortex rings at the edges of the shield, the droplets could enter the inside of the shield. In this case because the time when the droplets enter the inner surface of the shield is close to the time of the user’s inhalation (t = 0.25 s), the apparent probability that the user will inhale the droplets is high. To confirm this possibility, the trajectory of microparticles mixed into the sneezing flow was examined. Generally, the simulation of the trajectory of these particles considers the effects of gravitational forces, drag, droplet evaporation, droplet collapse, merger, and turbulent dispersion forces on particles.^25–28^ However, because the purpose of this simulation is to confirm the trajectory of the droplets when they travel with the flow, it was assumed that the target droplets were small enough to trace the flow (i.e., the mass of the particles is equal to zero). Furthermore, the Runge–Kutta method was used to estimate the trajectory of the droplets. This method is equivalent to using the fluid particles in a sneeze as a sample of droplets, which would be an overestimation of the actual trajectory of the droplets; thus, the worst case of droplet inhalation could be evaluated. Twenty particles were injected into the sneeze flow every 0.01 s at the inlet boundary that simulated the mouth of an infected person.

**FIG. 12.**
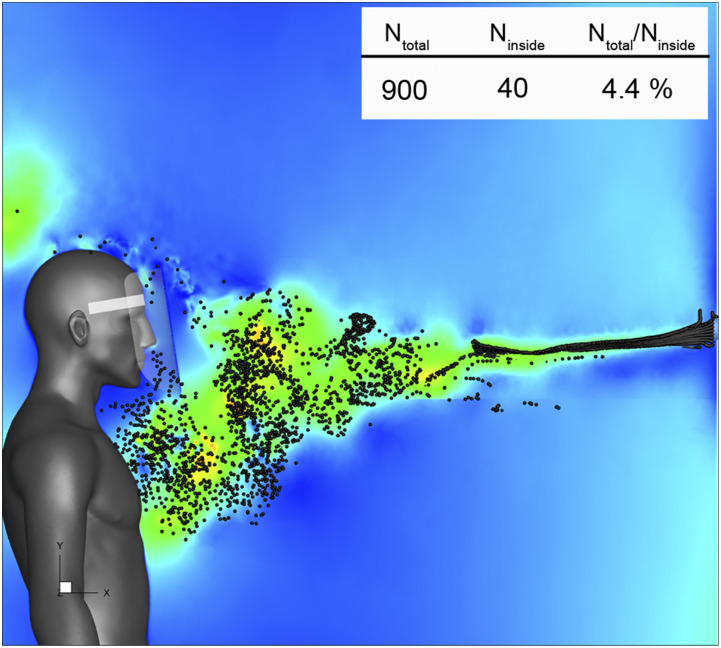
Distribution of aerosol-sized particles. Twenty particles were injected into the sneeze flow every 0.01 s at the inlet boundary that simulates the mouth of an infected person. N_total_ shows the total number of particles that were released. N_inside_ shows the number of particles that entered the inside of the face shield. Multimedia view: https://doi.org/10.1063/5.0031150.510.1063/5.0031150.5

Figure 12 (Multimedia view) depicts the results of particles spreading along the vertical cross section at t = 1.0 s when close to the end of the inhalation period. Some particles entered from the bottom edge of the face shield to the inside and reached close to the nose. This is because the leading vortex ring is generated immediately after the start of sneezing. At this time, the number of droplets is small; consequently, the number of particles captured in the vortex ring is considered to be small. Meanwhile, the trailing vortex rings were generated at a time when numerous particles were released and were floating along the trajectory; therefore, the number of particles captured in these vortex rings increased. Most of the particles that did not flow inside the shield reached the region between the neck and the chest of the shield user, indicating a high probability of droplet attachment in this area. The table in Fig. 12 shows the total number of particles that were released, the number of particles that entered the inside of the shield, and the ratio of both. In this simulation, 4.4% of the released droplets were found to enter the inside of the face shield and reached the vicinity of the nose. This result is approximately in agreement with the results of the experiments of Lindsley *et al.*, although the conditions of the tests are slightly different.^12^ It was confirmed that most of the particles that entered the inside of the shield were not caught by the entrainment flow generated by the vortex rings but by the inhalation of the breath with the particles that reached the bottom of the shield. It was also confirmed that some particles were inhaled through the nose. We consider that the possibility of inhalation of particles varies widely depending on the angle and distance between the shield wearer and the patient. This clarification is a subject for future work.

## IV. CONCLUSIONS

A flow analysis around the face shield was performed to examine the risk of virus infection when a medical worker wearing a face shield is subjected to an infected person’s sneeze from the front.

It was confirmed that the airflow in the space between the face shield and the face was observed to vary with human respiration. The high-velocity flow created by sneezing or coughing generates vortex ring structures, which gradually become unstable and deform in three dimensions. Vortex rings reach the top and bottom edges of the shield and form a high-velocity entrainment flow. It is suggested that vortex rings capture small-sized particles, i.e., sneezing droplets and aerosols, and transport them to the top and bottom edges of the face shield because vortex rings have the ability to transport microparticles. Droplets and aerosols from the patient’s sneeze are transported mainly by the trailing vortex rings moving downward in front of the face shield, while the leading vortex ring moving upward transports a relatively small number of droplets and aerosols. It was also confirmed that some particles (in this simulation, 4.4% of the released droplets) entered the inside of the face shield and reached in the vicinity of the nose. This indicates that a medical worker wearing a face shield may inhale the transported droplets or aerosol if the time when the vortex rings reach the face shield is synchronized with the inhalation period of breathing.

## DATA AVAILABILITY

The data that support the findings of this study are available from the corresponding author upon reasonable request.
